# Different Forms of Atrophy of the Teeth

**Published:** 1882-02

**Authors:** M. Parrot

**Affiliations:** Paris.


### ARTICLE VII.

### *On the Different Forms of Atrophy of the Teeth.*

BY M. PARROT, PARIS.

Extract from a paper entitled "Hereditary Syphilis as the Constant Cause of Rickets," read before the Section of Diseases of Children of the International Medical Congress, on Friday, Aug. 5th, 1881.

The effects of hereditary syphilis on the teeth, though less common than other signs of the disease, are far more lasting, persisting for centuries after the death of the individual, and bearing testimony to the great antiquity of syphilis. The lesions present themselves almost with mathematical accuracy, not only in the way in which the different teeth are affected, but also in their chronological relations. The different modifications, all of which may be classed under the common term of atrophy, are as follows:

1. Cupuliform atrophy.
2. Sulciform atrophy.
3. Atrophy of the cusps.
4. "Atrophie en hache" and
5. Hutchinson's atrophy.

The first, which is by far the most common form, especially attacks the permanent incisors. It is characterized by small superficial depressions, rarely isolated from each other, and almost always disposed in horizontal lines in the crown. The second variety appears in the same situation as the first, but in the form of parallel furrows. *Atrophy of the cusps* attacks all the teeth, but chiefly the first molars, and the permanent canines. It causes a division of the crown into two distinct but unequal portions. The portion furthest from the gum is smaller than normal in all its dimensions, is irregular, with pointed cusps, and seems, as it were, set in the lower portion. The above three varieties are often found in the same tooth; they are the result of pathological modifications of the dentine and enamel, and date from the interalveolar period of dental development.

The fourth and fifth varieties, on the other hand, do not make their appearance until after the eruption of the teeth, and are the result of caries or derition of parts predisposed to these changes by reason of a congenital alteration in the enamel. "*Atrophie en hache*" only occurs in the upper incisors. For a certain time the portion nearest to the gum is only eroded, and as the cutting edge remains intact, the appearance of a steel hatchet is produced. In *Hutchinson's atrophy*, on the contrary, the part attacked is the central portion of the cutting edge of the incisors which is rapidly worn away owing to the thinning of the enamel. Hence results a notch of variable depth, and triangular or crescentic in shape.

Atrophy may attack all the teeth with the exception of the second and third molars and the permanent bicuspids.

Its existence in patients, the subject of hereditary syphilis, together with the coincident between the time of its evolution and the active period of that affection, entitle me to refer it to that cause and authorize me in refusing to accept —if not absolutely, at least in the vast majority of cases— any other etiology. As a matter of fact the infantile pyrexiæ, of which so much has been said, do not as a rule make their appearance until after the second year, when the formation of the teeth is already complete; and as for the convulsions which, in the eyes of M. Magitot and his pupils, play such an exclusive part in the etiology of eron, I reject them altogether. First, because in the majority of cases the alteration has been produced or at any rate has commenced during intra uterine life, the neuropathic accidents of which are unknown; secondly, because the intensity and the depth of the lesions, while accurately corresponding to the duration and intense action of hereditary syphilis, cannot be made to square with the relatively short period during which the eclamptic attacks occur; and finally, because the two last molars are never attacked, a phenomenon easy of explanation, on my theory, the activity of the syphilitic poison having spent itself by the time these teeth are developed, but entirely contradictory to the opinion of M. Magitot, since convulsions are far from being rare at that period of infantile life.—*British Journal of Dental Science.*

